# Gene and functional up-regulation of the BCRP/ABCG2 transporter in hepatocellular carcinoma

**DOI:** 10.1186/1471-230X-12-160

**Published:** 2012-11-15

**Authors:** Caecilia HC Sukowati, Natalia Rosso, Devis Pascut, Beatrice Anfuso, Giuliano Torre, Paola Francalanci, Lory S Crocè, Claudio Tiribelli

**Affiliations:** 1 Centro Studi Fegato, Fondazione Italiana Fegato, Bld Q AREA Science Park Basovizza, Trieste, Italy; 2 Ospedale Pediatrico Bambino Gesù, Rome, 00100, Italy; 3 Department of Medicine, University of Trieste, Trieste, 34100, Italy

**Keywords:** Liver cancer, Drug resistance, Gene expression, Doxorubicin

## Abstract

**Background:**

The Breast Cancer Resistance Protein (BCRP/ABCG2) is one member of ABC transporters proteins super family responsible of drug resistance. Since data on ABCG2 expression in liver malignances are scanty, here we report the expression of ABCG2 in adult human hepatocellular carcinoma (HCC) in both *in vivo* and *in vitro* models with different degree of malignancy.

**Methods:**

In cell lines derived from human hepatocellular carcinoma, ABCG2 gene expression was assessed by reverse transcription quantitative real time PCR and function by Hoechst 33342 efflux assay; protein content was assessed by SDS-PAGE Western blot.

**Results:**

ABCG2 expression was found to be highest in the most undifferentiated cell lines, and this was related with a higher functional activity. ABCG2 expression was sensitive to antineoplastic drugs since exposure to 5 μM doxorubicin for 24 hours resulted in significant up-regulations of ABCG2 in all cell lines, particularly in those lines with low basal ABCG2 expression (p<0.01). The gene expression was also investigated in 51 adult liver tissues with HCC and related cirrhosis; normal liver tissue was used as control. ABCG2 gene expression was higher in HCC than both cirrhotic paired tissue and normal tissue. This up-regulation was greater (p<0.05) in pathological poorly differentiated grade G3/G4 than in well-differentiated G1/G2 HCC.

**Conclusions:**

Our results suggest a correlation of ABCG2 gene expression and differentiation stage both in human and HCC derived cell lines. The rapid up-regulation of ABCG2 to exposure to doxorubicin emphasizes the importance of this transporter in accounting for drug resistance in liver tumors.

## Background

Primary liver cancer is one of the most common cancers in the world and the third cause of cancer-related death [1]. Hepatocellular carcinoma (HCC) accounts for around 85-95% of all PLC cases. Targeted systemic therapies to treat HCC patients with advanced stages had become one of urgent needs in the management of HCC [2]. However, systemic therapies also have been faced many obstacles, such as drug toxicity and drug resistance.

The Breast Cancer Resistance Protein (ABCG2/BCRP/MXR) is one member of the ATP-Binding Cassette (ABC) transporters superfamily proteins [3]. One of the key functions of ABC transporter is related with the cell resistance to exposure of external compounds, exporting the drug out of the cells thus maintaining the intracellular drug compound below toxic level. ABCG2 protein is widely expressed in tissues, mainly in placenta, epithelium of small intestine and colon, liver canalicular membrane and breast [4]. Furthermore, ABCG2 is also expressed in the epithelium of the prostate and bladder, endocervical cells of uterus, kidney tubules and others tissues [5].

The association between ABCG2 and cancer has been extensively studied. The ABCG2 protein was observed in a wide variety of tumors [6] and shown to be involved in the cancer cells resistance to many chemotherapeutic agents [7,8]. However, even though there are several reports of ABCG2 in liver cancer [9-12], it is still needed to be expanded and its correlation with clinical finding is important to be explored. The ABCG2 expression was found to be high in HCC [10] and was reported to increase following chemotherapy in hepatoblastoma patients [13]. In glioma, ABCG2 expression is related with pathological grade, tissues differentiation and resistance to mitoxantrone [14].

In recent studies, ABCG2 expression has been also associated with stem cells and cancer stem cells, both in circulating and solid cancers. Side population (SP) of stem cells is a population of cells having the ability to export Hoechst 33342 dye, an activity related with cells resistance [15]. In HCC, the SP cells sorted from several cell lines were associated with the metastatic potentials and therapeutic-resistance [16]. Zhou *et al.* demonstrated that the ABCG2/Bcrp1 is the molecular determinant for SP [17,18] and SP of hepatic oval cells was also defined by ABCG2/BCRP1 in the rat model [19]. Furthermore, cells expressing ABCG2 might play a central role in hepatocarcinogenesis, in which ABCG2^+^ cells could generate both ABCG2^+^ and ABCG2^-^ cells, whereas ABCG2^-^ cells bore only ABCG2^-^ cells [20].

Collectively these studies indicated the relevance of ABCG2 in several human malignancies and its association with drug resistance and cells differentiation. We here report on the ABCG2 mRNA level and activity in both *in vitro* and *in vivo* models consisting of human hepatic cell lines and human samples of HCC to investigate the role of this transporter, particularly its role in drug resistance issue in liver cancer.

## Methods

### Samples

#### Cell lines

Human liver cell lines IHH, HepG2, HuH-7 and JHH-6 were used as *in vitro* models of HCC cell lines with different degree of morphologic differentiation. The immortalized hepatocyte line IHH was kindly provided by Dr. Trono (Lausanne, Switzerland) [21] while human HCC cell lines HuH-7 (JCRB0403) and JHH-6 (JCRB1030) were obtained from the Japan Health Science Research Resources Bank (HSRRB, Tokyo, Japan). The HepG2 cell line was obtained from the Istituto Zooprofilattico Sperimentale della Lombardia e dell’Emilia Romagna (IZSLER, Brescia, Italy).

The IHH cells were grown in DMEM-F12 medium supplemented with 10% (v/v) fetal bovine serum (FBS), 1% antibiotics, 1% L-glutamine, 1 μM dexamethasone and 5 μg/mL insulin. The HepG2 and HuH-7 cells were grown in DMEM medium (high glucose) supplemented with 10% (v/v) FBS, 1% L-glutamine and 1% antibiotics. The JHH-6 cells were grown in Williams’ E medium supplemented with 10% (v/v) FBS, 1% L-glutamine and 1% antibiotics. The cultures were maintained at 37°C in a humidified 5% CO_2_ incubator and when they reached 80% - 90% confluence they were routinely expanded by 0.05% trypsin detachment.

#### Human tissue samples

Human liver tissues were collected from HCC patients undergoing liver resections or orthotopic liver transplantations and normal donor liver in the similar age group as control. A total of 51 (23 HCC, 21 cirrhosis, 7 normal tissues) liver tissues were analyzed. Fifteen paired samples HCC and cirrhosis were obtained from the same patient (70% of all HCC tissues analyzed). The tissues were snap-frozen in liquid nitrogen and stored in −80°C before further processing. The diagnosis of patients was established on international criteria together with its Edmondson Steiner HCC grading [22] and other clinical findings. Informed consent to participate to the study was obtained from each patient or by a legal representative and the protocol was approved by the ethical committee of the University of Trieste.

### *In vitro* cytotoxicity test

The cytotoxic effects of doxorubicin hydrochloride (Dox), verapamil hydrochloride, Hoechst 33342 (Sigma-Aldrich, St Louis, USA) and Rhodamine 123 (Rho123; Invitrogen, Milan, Italy) were assessed by 3-(4,5-Dimethyl-2-thiazolyl)-2,5-diphenyl-2H-tetrazolium bromide (MTT) dye reduction test [23]. The cells were seeded in concentration 20,000 cells/cm^2^ in 24-well plates for the corresponding time. The dose of Dox, verapamil, Hoechst, and Rho123 ranged from 0 to 10.0 μM, 0 to 20 μM, 0 to 5 μg/mL, and 0 to 20 μg/mL, respectively. For Dox cytotoxicity test, the exposure time was 24 hours, whereas for verapamil, Hoechst and Rho123 the exposure time was tested at 30, 90 and 180 minutes as the required time to perform the dye exclusion assay. The absorbance of the untreated cells was taken as 100% survival.

### Total RNA isolation and reverse transcription

Total RNA from cell lines and tissues samples was extracted using the TriReagent solution (Sigma–Aldrich) according to the manufacture’s protocol. The RNA pellet was dissolved in nuclease-free water and stored at −80° C until further analysis. RNA was quantified at 260 nm in a Beckman Coulter DU®730 spectrophotometer (Fullertone, CA, USA). The RNA purity was evaluated according the MIQE guidelines [24] by measuring the ratio A260/A280 with appropriate purity values between 1.8 and 2.0. The integrity of RNA was assessed on standard 1% agarose/formaldehyde gel. The reverse transcription of 1 μg of total RNA was performed with an iScript cDNA synthesis Kit (Bio-Rad, Milan, Italy) according to the manufacture’s suggestions. A total of 20 μL volume reaction was conducted in a thermocycler (Gene Amp PCR System 2400, Perkin-Elmer, Boston, MA, USA) at 25°C for 5 min, 42°C for 30 min, 85°C for 5 min. The cDNA was conserved at −20°C until used.

### Quantitative real time RT-PCR (RT-qPCR)

The RT-qPCR of gene ABCG2 and ABCB1 was performed according to the iQ SYBR Green Supermix protocol (Bio-Rad). PCR amplification was carried out in 15 μL reaction volume containing 25 ng of cDNA, 1x iQ SYBR Green Supermix (100 mM KCl; 40 mM Tris–HCl; pH 8.4; 0.4 mM each dNTP; 50 U/mL iTaq DNA polymerase; 6 mM MgCl_2_; SYBR Green I; 20 mM fluorescein; and stabilizers) and 250 nM gene specific sense and anti-sense primers (Table 1). Reactions were run and analyzed on a Bio-Rad iQ5 multi color real-time PCR detection system (IQ5 software version 3.1) together with reference genes. Cycling parameters were determined and analyzed using the Pfaffl modification of the ΔΔCt equation with taking accounts to the efficiency of the reaction [25]. Primer sets were designed using the Beacon Designer 7.9 (Premier Biosoft International, Palo Alto, CA, USA) across two exons to avoid contamination of genomic DNA and reference genes 18S-rRNA and β-actin were used to normalize target gene expression. Melting curve analysis and agarose gel electrophoresis were carried out to asses templates specificity.

**Table 1:** List of primer sequences for the quantification of specific genes by RT-qPCR in hepatic cell lines and clinical samples tissues

**Gene**	**Acc. no.**	**Primer forward**	**Primer reverse**	**Ref.**
ABCG2	NM_004827	TATAGCTCAGATCATTGTCACAGTC	GTTGGTCGTCAGGAAGAAGAG	This study
ABCB1	NM_000927	TGCTCAGACAGGATGTGAGTTG	AATTACAGCAAGCCTGGAACC	This study
β-actin	NM_001101	CGCCGCCAGCTCACCATG	CACGATGGAGGGGAAGACGG	This study
18S-rRNA	NR_003286	TAACCCGTTGAACCCCATT	CCATCCAATCGGTAGTAGCG	[26]

### Western blot

After 5 μM Dox treatment for 24 hours, cells were washed with PBS at room temperature. The protein was extracted using cell lysis buffer (PBS containing 1% v/v of a protease inhibitor cocktail and 2 mM phenylmethylsulfonylfluoride). Protein concentration was determined by copper (II) sulphate solution and bicinchonic acid (Sigma-Aldrich) protein assay following the manufacturer’s instructions.

A total of 30 μg protein were size-separated, together with molecular weight standard by SDS–PAGE using a Mini Protein III Cell (Bio-Rad, Hercules, CA, USA). After SDS–PAGE, proteins were electro-transferred with a semi-dry blotting system onto immune-blot PVDF membranes using a Mini Trans-Blot Cell (Bio-Rad). Membrane was incubated overnight at 4°C with targeted antibodies in T-TBS buffer (Tris 20 mM, Tween 20, 0.2%, NaCl 500 nM, pH 7.5). The peroxidase reaction was obtained by exposure of membrane in the ECL-Plus Western blot detection system solutions (ECL Plus Western blotting Detection Reagents, GE-Healthcare Bio-Sciences, Italy). List of antibodies was described in Table 2.

**Table 2:** List of antibodies

**Antibody**	**Clone**	**MW kDa**
ABCG2/BCRP	BXP53 (Abcam, Cambridge, MA, USA)	72
ABCB1/MDR1	C219 (Abcam, Cambridge, MA, USA)	170
Actin	A2066 (Sigma-Aldrich, St. Louis, MO, USA)	42
Anti rabbit IgG peroxidase	P0448 (Dako, Glostrup, Denmark)	
Anti rat IgG peroxidase	P0450 (Dako, Glostrup, Denmark)	
Anti mouse IgG peroxidase	P0260 (Dako, Glostrup, Denmark)	

### Hoechst 33342 efflux assay

The activity of ABCG2 in cell lines IHH, HepG2, HuH-7 and JHH-6 was assessed using Hoechst 33342 efflux assay with modification from previous report [27]. Single cells suspension and monolayer cells were given sterile 1 to 20 μM final concentration of verapamil incubated for 30 minutes in 37°C. After incubation, 5 μg/mL Hoechst 33342 was added, and the cells incubated for 90 minutes in 37°C. The reaction was stopped by incubating the cells on ice for 5 minutes. The Hoechst 33342 efflux was measured by spectrofluorometer (Jasco FP-770, Maryland, USA and Hidex Chameleon - Driver 4.34, Turku, Finland) for suspension and monolayer cells, respectively. The measurement was performed on 355 nm excitation and 460 nm emission wavelength. To confirm the ABCB1 activity, the internal accumulation of Rho123, was performed by FACSCalibur flow cytometer (Beckton Dickinson, NJ, USA). A total of 10,000 events were analyzed per sample.

### Statistical analysis

Box plot graphics and statistical analysis were constructed using software SigmaPlot Version 11.0 (Systat Software, Inc., Chicago, USA). The student’s *t* test was performed for statistical comparison between groups. Value of p<0.05 was regarded as statistically significant.

## Results

### Modulation of the ABCG2 gene and protein expression by doxorubicin

**Figure 1:**
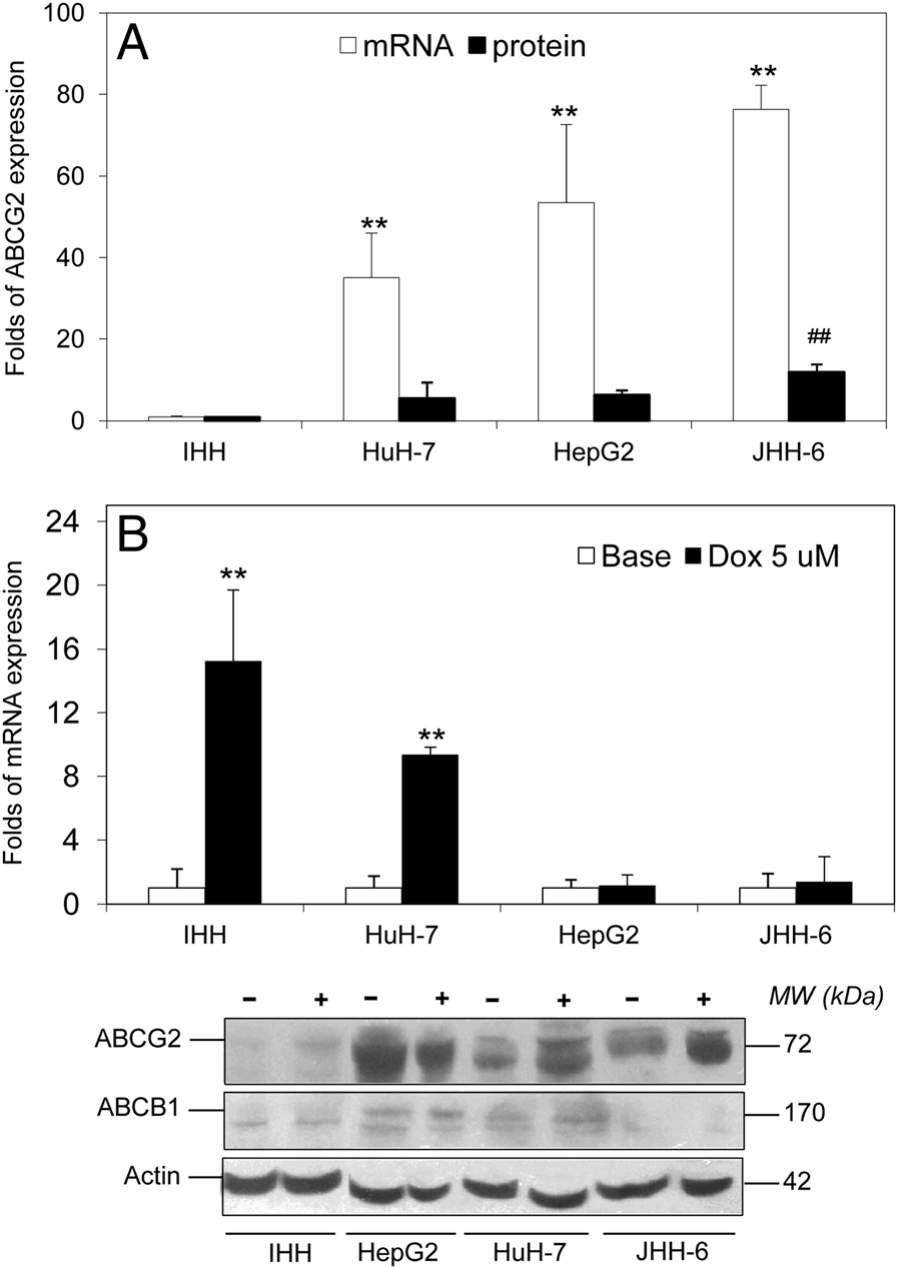
**The expression of ABCG2 in hepatic cell lines. A**. The basal ABCG2 mRNA and protein expression in human hepatic cell lines IHH, HuH-7, HepG2 and JHH-6. The data represented the mean ± SD of minimum three independent experiments. Statistical student’s *t* test: mRNA **p<0.01 to IHH, protein ^##^p<0.01 to IHH. **B**. The folds of ABCG2 mRNA up-regulation after exposure to 5 μM Dox for 24 hours compared to basal expression considered (1.00). Statistical students’ *t* test: ** p<0.01 to basal expression of each cell lines. The mRNA expression was normalized to reference genes 18S-rRNA and β-actin. Protein blot showing a representative Western Blot of ABCG2 (72 kDa) and ABCB1 (170 kDa) protein. Symbol: – control (basal level), + Dox.

To investigate the ABCG2 expression according to cell differentiation, 4 human-derived hepatic cell lines with different degree of differentiation were analyzed. The expression of immortalized normal human hepatocytes IHH was defined as control 1.00. The highest level of ABCG2 folds mRNA expression compared to IHH was observed in the most undifferentiated cells JHH-6 (76.27 ± 6.00), followed by the more differentiated cells HepG2 (53.52 ± 19.06) and HuH-7 (35.07 ± 10.96) with p<0.01. In line with the mRNA results, ABCG2 protein (72 kDa) was detected in tumoral cell lines HuH-7, HepG2, and JHH-6, but not or very weak in IHH (Figure 1A). The protein expression in JHH-6 is 11.5 ± 1.5 folds higher compared to IHH (p<0.01).

We then assessed the effect of ABCG2 expression after exposure to Dox. When exposed to 5 μM Dox for 24 hours, 50% cell population in all cell lines tested died. In the surviving cells of each cell line, the exposure to Dox induced an up-regulation of ABCG2 mRNA. Of notice the extent of the up-regulation was inverse to the basal level of expression as IHH and HuH-7 showed a significant increment of gene expression (15.25 ± 4.45 and 9.36 ± 0.48 folds, respectively, p<0.01) as compared to HepG2 and JHH-6 which the expression was unchanged (1.14 ± 0.68 and 1.38 ± 1.58 folds, respectively). In line with the gene expression, ABCG2 protein was weakly detected in IHH cells at basal condition, but it became detectable after exposure to Dox. The same occurred in HuH-7 while no difference was observed in JHH-6 cells where the treatment did not induce a significant over-expression of the transporter. However, even though there was a striking ABCG2 up-regulation compared to their basal level, the level of ABCG2 in IHH after Dox did not reach the basal level of HuH-7, HepG2, and JHH-6, still about a half of basal HuH-7 expression (Figure 1B).

### The functional activity of ABCG2

To define the activity of the ABCB1 and ABCG2 protein in the cell lines, the Hoechst 33342 efflux assay was determined. Preliminary experiments showed no effect on cell viability (MTT test) by verapamil, Hoechst and Rho123, to all cell lines, on working concentration of 10 μM, 5 μg/mL, and 10 ug/mL (96.6 ± 5.3%, 90.6 ± 7.4%, and 94.9 ± 8.1%, respectively). As control, we evaluated also the level of basal ABCB1 expression in these cell lines. The ABCB1 mRNA expression in IHH and JHH-6 were really low, more than 1000 times lower compared to those of HuH-7 and HepG2. ABCB1 protein expression was shown in Figure 1B.

As shown in Table 3, the increase of Hoechst concentration (2.5 – 10.0 μg/mL) increased the activity of these two transporters to export dyes. This was defined by the decrease of Hoechst intracellular dye accumulation. These evidences showed the capacity of both transporters to efflux the dyes out of the cells as a mechanism of cells toxicity defense mechanism.

**Table 3:** The efflux capacity of the HCC cell lines in exporting Hoechst 33342

**HCC lines**	**Hoechst 33342 (μg/mL)**		
	2.5	5.0	10.0
HuH-7	100.0 ± 6.4	88.4 ± 5.5	53.0 ± 2.8
HepG2	100.0 ± 6.2	74.7 ± 3.1	51.2 ± 2.8
JHH-6	100.0 ± 5.8	80.1 ± 2.1	53.7 ± 4.0

The increase of Hoechst 33342 concentration decreased intracellular dye accumulation in all HCC cell lines tested. The intracellular dye intake of 2.5 μg/mL as lowest dye concentration was considered as 100.0%. The data represented the mean ± SD of three independent experiments.

**Figure 2:**
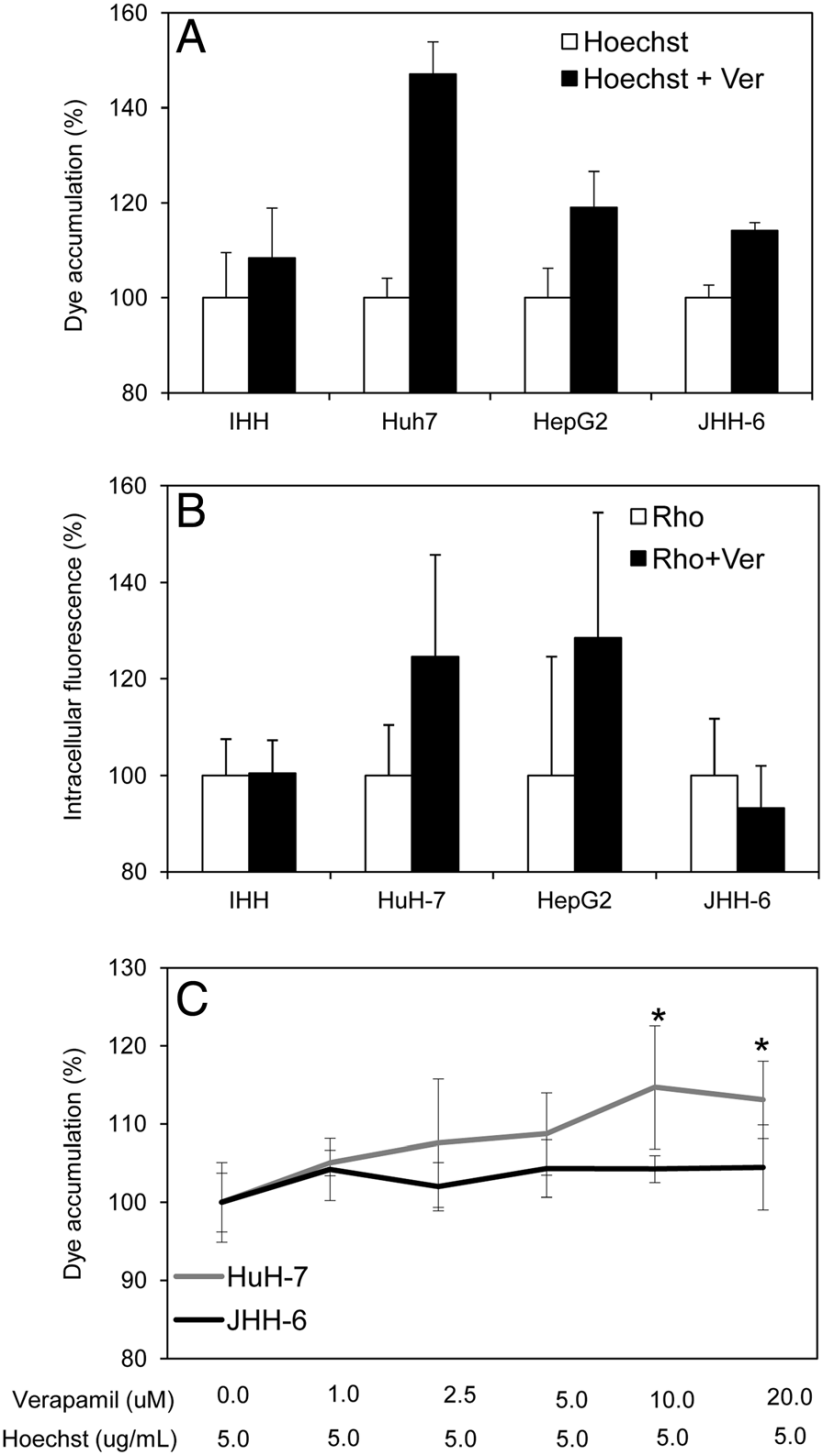
**Functional capacity of ABCG2. A**. The uptake of intracellular Hoechst 33342 accumulation measured by spectrofluorometry. The difference in intracellular dye accumulation with and without ABCB1 inhibitor verapamil was observed to be lower in JHH-6 followed by HepG2 and HuH-7 indicating major functional activity of ABCG2 in JHH-6. Cells without verapamil = 100.0%. **B**. Representative histogram plots showing the intracellular fluorescence of Rho123 in 10,000 events by FACS analysis. The data represented the mean ± SD of minimum three independent experiments. **C**. The dose-dependent intracellular Hoechst 33342 accumulation in monolayer HuH-7 and JHH-6 cells. Statistical student’s *t* test: *p<0.05.

Since the extracellular extrusion Hoechst 33342 is accounted by two ABC proteins (ABCB1 and ABCG2), to establish the relative role of ABCG2 the assay was also performed in the presence of verapamil, a potent ABCB1 but a weak ABCG2 inhibitor [30]. In the HCC cell lines which have both ABCB1 and ABCG2, the lowest differential Hoechst fluorescence signal between cells with and without verapamil was found in cells JHH-6, indicating the ABCG2 in this cell line is dominantly responsible of the dye efflux. The result of Rho123 efflux measurement by FACS confirmed the functionality of ABCB1 was observed higher in HuH-7 and HepG2 (Figure 2A). A dose dependent Hoechst 33342 internal dye accumulation with the presence 1 to 20 μM verapamil in monolayer cells gave similar result (Figure 2B).

### ABCG2 expression *in vivo*

**Figure 3:**
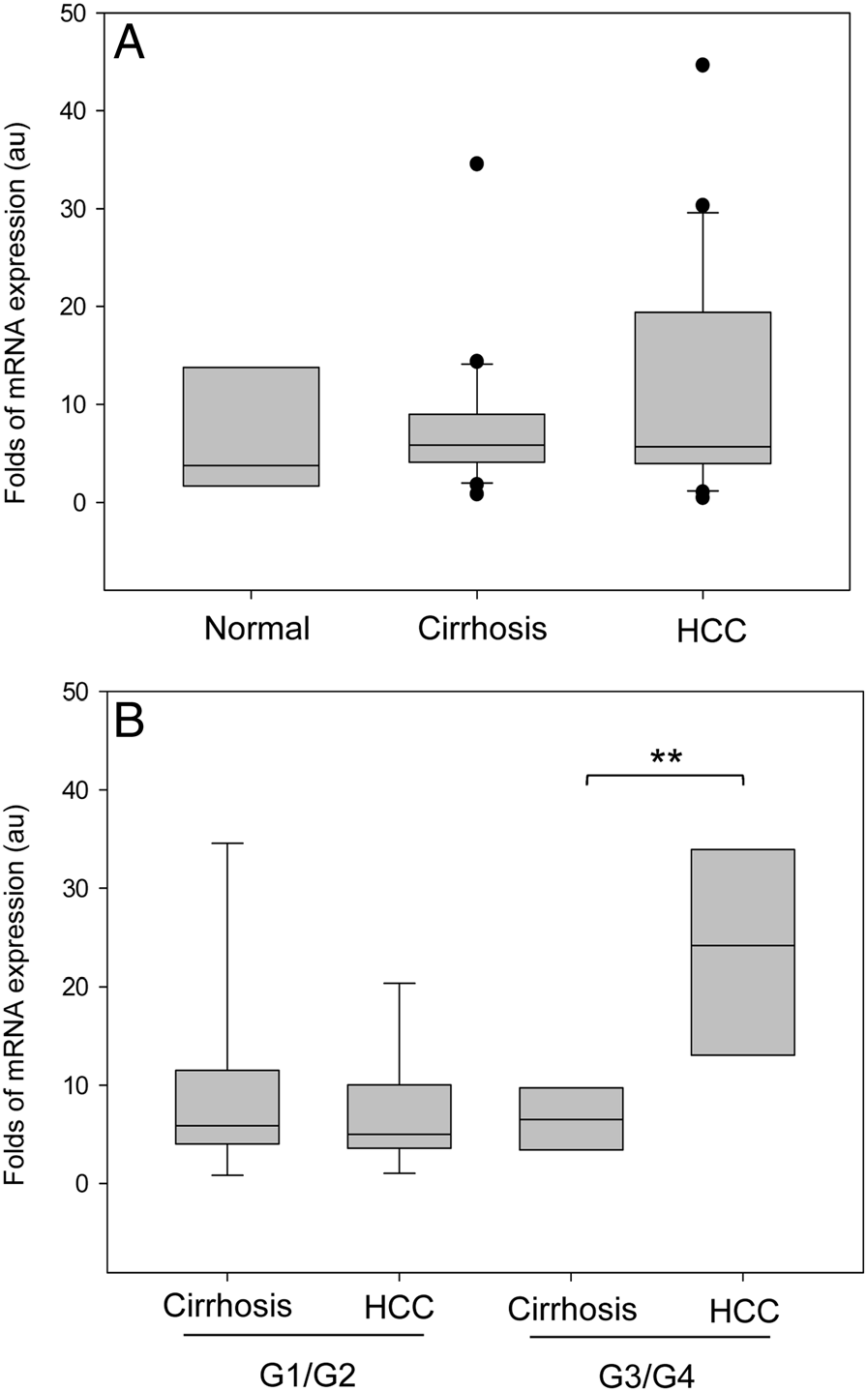
**The expression of ABCG2 mRNA in HCC tissues. A**. The general distribution of ABCG2 mRNA expression in HCC and normal liver. Normal liver (N; n = 7), cirrhotic tissue of HCC (n = 21), and tumoral tissue of HCC (n = 23). **B**. The up-regulation of ABCG2 mRNA in the HCC compared to its cirrhotic paired tissues from 15 paired individuals with histological G1/G2 and G3/G4 grade. The mRNA expression was normalized to reference genes β-actin and 18S-rRNA. A normal liver was considered as 1.0 au. Statistical student’s *t* test: ** p<0.01.

To confirm the data found in the cell lines in an *in vivo* series, we measured the quantity of ABCG2 mRNA in human samples collected from patient with HCC and cirrhosis. The ABCG2 mRNA levels in the 51 tissues samples were estimated by RT-qPCR and expressed in arbitrary units (au); a sample obtained from normal human liver was considered as 1.00 au. As shown in Figure 3A, ABCG2 expression was found to be rather variable in all groups and in particular in HCC patients. The ABCG2 expression were found to be more restricted in normal and cirrhotic tissues compared to HCC

When HCC patients were divided according to the Edmondson Steiner HCC grading system, the expression of ABCG2 was found to be higher in HCC with poorly differentiated HCC G3/G4 (n=6) as compared to well- and moderately-differentiated HCC G1/G2 (n=13) (p<0.05). In 15 paired samples, the up-regulation of ABCG2 in HCC compared to its cirrhotic tissue was consistently found to be higher in G3/G4 HCC (ratio 3.24 ± 2.08 folds) than in G1/G2 HCC (ratio 1.25 ± 1.06 folds) (p<0.01) as shown in Figure 3B. This significance was not observed for ABCB1 gene. There was no correlation of ABCG2 with other clinical findings such as MELD and CTP score, etiology, alpha fetoprotein and albumin level, and vascular invasion.

## Discussion

The non-responsiveness of cancer cells to chemotherapeutic regiments has been a major obstacle in the treatment of cancers. The survival rate for HCC patients in advanced stages which could not treated with surgical procedures (including liver transplantation) has not been changed for 30 years [28]. One of the important issues in multidrug resistance in cancer is the high expression of ABC transporters proteins, including ABCG2.

Due to the inconclusive data on the role of ABCG2 in HCC we assessed the functional and gene expression of this ABC transporter in two different but related systems such as a series of human derived liver cell lines and patients with HCC and cirrhosis. For the *in vitro* model 4 differently differentiated hepatic cell lines were used. Three derived from HCC while one originates from a normal adult liver. The ABCG2 mRNA expression in tumoral lines was higher than in immortalized normal hepatocyte (IHH) and this was paralleled by the protein contents. Of notice was the effect observed when the cells were exposed to 5 μM Dox. This treatment induced an up-regulation of both gene expression and protein content in all cell lines, but the up-regulation was inversely proportional to the basal level of expression. This behavior suggests that in the presence of toxic drug, ABCG2 is induced to export the drug from the cell and to prevent cytotoxicity as one of the major defense mechanisms and that this effect is maximal when the basal level of the expression is low. The data we observed in HepG2 cells agree with a previous report in which ABCG2 mRNA was up-regulated in Dox-resistant HepG2 [29].

To assess the activity of ABCG2 in cancer cell lines, we performed a functional assay using the Hoechst 33342 test. The efflux of Hoechst 33342 is accounted by both ABCG2 and ABCB1 transporters, and verapamil had been shown to effectively inhibit ABCB1 but not ABCG2 [30]. The addition of verapamil resulted in the increase of intracellular Hoechst 33342 in the well differentiated HepG2 and HuH-7 cells but not in the poorly differentiated cells (JHH-6). This data points to the conclusion that in the poorly differentiated HCC cell line, the efflux is mainly depend on the ABCG2 activity while in the more differentiated ones, drug export is also accounted by the activity of ABCB1. This conclusion is in line with very high ABCB1 expression in both HepG2 and HuH-7 cells. This finding indicates that a high expression of ABCG2 transcription is associated with functional role of the transporter which results in the protection of the cell from doxorubicin-induced cytotoxicity.

To compare the data obtained *in vitro*, we studied the ABCG2 mRNA expression in samples of adult normal liver and tissues obtained from HCC patients. There are several limitations in this type of studies such as the effect of the different treatments to which each patient undergoes before being considered for hepatic resection or transplantation. Our data in human tissues samples show a rather high variation in ABCG2 expression, as expected. The large variations of intra- and inter-groups ABCG2 expression observed in this study had also been reported in acute leukemia [31], breast cancer [32] and lung cancer [33]. We believe that these variations are likely linked to several factors such as age, sex, type of the drugs, duration of the treatments, severity of the diseases, and many others. In line with this conclusion is the observation that when we compared the expressions of normal adult liver with that obtained from children, ABCG2 expression was higher in the adults (data not shown), suggesting that ABCG2 expression is age-related and dependent to drug exposure which obviously increases with age.

The overall distribution showed that the expression of the ABCG2 mRNA was slightly higher and more dispersed in HCC and cirrhotic tissues as compared to normal tissues, in line with a previous report [10]. Interestingly, the ABCG2 mRNA expression was higher in the HCC than in the surrounding cirrhotic tissue in about 60% of HCC patients. Of notice was the observation that this up-regulation was consistently observed in poorly differentiated pathological grade G3/G4 than in G1/G2 HCC tissue. This is different from what described by Gupta *et al.* who reported in 3 specimens of liver cancers that both the mRNA and protein level of ABCG2 were decreased [34]. The discrepancy suggests that the ABCG2 expression is variable and dependent by the tissue type, even in the single individual due to variability of HCC tissues profile. A relationship between cell differentiation and ABCG2 expression had been previously reported in other cell lines. A high level of functional ABCG2 was detected in undifferentiated human embryonic cells and decreased during cellular differentiation [35]. In hematopoietic system, the ABCG2 expression is confined to the most immature progenitor cells and down-regulated at the committed progenitor level [36]. Recent data showed that knock-down of ABCG2 inhibited breast cancer and lung cancer cells proliferation, suggesting the role of ABCG2 in maintenance the cancer cells [37].

Our finding reemphasized the importance of intrinsic factors like age, differentiation, and disease status in the expression of drug-transporters in human tissues. Our data expand the previous report showing that ABCG2 protein and mRNA were higher in HCC [10] and that in hepatoblastoma patients the transporter was up-regulated following chemotherapy [13]. For the ABCG2 and pathological grade of cancer, our data in HCC is concordant with those observed in glioma [14].

## Conclusions

Our findings demonstrated an higher expression of the ABCG2 in liver diseases compared to normal tissue and possibly more important, in HCC where the genetic and functional up-regulation is more marked in less differentiated tumors. The up-regulation of ABCG2 observed in response to drug therapy underlines the importance of this transporter to account for the drug resistance and points the needs to explore its expression in liver diseases, HCC in particular.

## Competing interests

The authors declare that they have no competing interests.

## Authors’ contributions

CHCS conducted the experiments and wrote the article; NR, DP, and BA performed experiments; PF, GT, and LSC read and approved the text; CT read, edited and approved the text. All authors read and approved the final manuscript.

## Pre-publication history

The pre-publication history for this paper can be accessed here:

http://www.biomedcentral.com/1471-230X/12/160/prepub
